# What do people know about fertility? A systematic review on fertility awareness and its associated factors

**DOI:** 10.1080/03009734.2018.1480186

**Published:** 2018-06-29

**Authors:** Juliana Pedro, Tânia Brandão, Lone Schmidt, Maria E. Costa, Mariana V. Martins

**Affiliations:** a Faculty of Psychology and Education Sciences, University of Porto, Porto, Portugal; b Centre for Psychology, University of Porto, Porto, Portugal; c Centre for Research in Psychology (CIP-UAL), Universidade Autónoma de Lisboa, Lisboa, Portugal; d Department of Public Health, University of Copenhagen, Copenhagen, Denmark

**Keywords:** Age-related fertility decline, fertility awareness, health-literacy, infertility risk factors, obesity, sexual transmitted infections, smoking, systematic review

## Abstract

**Introduction:**

Recent evidence indicates that reproductive-age people have inadequate fertility awareness (FA) concerning fertility, infertility risk factors, and consequences of delaying childbearing. However, no study has tried to summarize these studies and to clarify the variables associated with FA, namely the role of gender, age, education, and reproductive status on FA.

**Methods:**

A literature search up to February 2017 was conducted using the EBSCO, Web of Science, Scielo, and Scopus electronic databases with combinations of keywords and MeSH terms (e.g. ‘awareness’ OR ‘health knowledge, attitudes, practice’ AND ‘fertility’; ‘fertile period’; ‘assisted reprod*’).

**Results:**

Seventy-one articles met the eligibility criteria and were included. The main results showed that participants report low-to-moderate FA. Higher levels of FA were shown by women, highly educated individuals, people who reported difficulties with conceiving, and those who had planned their pregnancies. Having or desiring to have children was not related to FA level. An inconsistent association between study participant age and FA was observed, with some studies indicating that older participants had higher FA, but others found an opposite result or did not find any association.

**Conclusion:**

The current findings suggest that interventions to increase FA are warranted, especially those targeting men, people with low education, and in family planning settings. Interventions and campaigns should be customized to meet individuals’ needs regarding FA. Because of the high heterogeneity regarding the assessment of FA, these conclusions must be interpreted with caution.

## Introduction

‘Beauty has no age. Fertility does’. These sentences were one of the slogans used in 2016 to mark Fertility Day in Italy. Although this campaign was highly controversial, it reflects current concerns regarding the trend of postponing childbearing to later ages (1) and the lack of sufficient fertility awareness within the reproductive-age population.

The determinants and consequences of delaying pregnancy have been analysed and discussed by gynaecologists, public health experts, psychologists, demographers, and politicians (2–5). Studies have shown that the decision to have children is multifaceted and determined not only by individual, social, and economic factors but also by social policies (2,3,5). Hence, delaying parenthood is not always a conscious process (6), and it seems to make an important contribution to the incidence of infertility (7). Infertility is a public health problem (8,9) that affects people around the world. Although not all infertility problems can be prevented, some important risk factors are preventable (10). Advanced female age is related to fewer and poorer-quality follicles (11,12) as well as a higher risk of miscarriage (12–14), obstetric morbidity, and perinatal complications (15). Female and male ages are both associated with an increased time to pregnancy (16,17). A recent study estimated that if a couple desires to have two children without making use of fertility treatments, they should start trying to conceive when the woman is 27 years old to have a 90% chance of success (18).

In addition to age, other modifiable factors include sexually transmitted infections (STIs) (19), smoking (20,21), alcohol consumption (22,23), and obesity or low weight (21,24,25); for a full review of these and other risk factors, see (26–28). Further, in less developed countries unsafe abortions, pregnancy-related infections, and insufficient delivery care are important risk factors for secondary infertility (29). In addition, some myths and misconceptions concerning fertility, reproduction, and fertility treatments remain, which might delay help-seeking behaviours and negatively affect reproductive plan management (30,31).

Together with the increasing evidence regarding infertility risk factors, the trend towards childbearing postponement has stimulated researchers to assess fertility awareness (FA)—a concept recently defined in The International Glossary on Infertility and Fertility Care as ‘the understanding of reproduction, fecundity, fecundability, and related individual risk factors (e.g. advanced age, sexual health factors such as sexually transmitted infections, and life style factors such as smoking, obesity) and non-individual risk factors (e.g. environmental and work place factors); including the awareness of societal and cultural factors affecting options to meet reproductive family planning, as well as family building needs’ (32). A majority of studies focused on FA suggests that young adults of reproductive age desire to have children (33,34) but they are not sufficiently informed about age-related fertility decline and infertility risk factors (33,35–37). This lack of knowledge led to the emergence of public FA campaigns. However, some of these campaigns were not well accepted by the public, with media reactions stating that the reproductive-age population felt pressure to have (more) children (e.g. ‘Advancing age decreases your ability to have children’, *USA Seattle News and Events*, 9 October 2006; ‘Even the best marksman could miss the target’, *Strait Times*, 5 February 2016; ‘Swimming too slowly?’, *Independent*, 2 June 2016 (38–40)). Consistent with this finding, education programmes have revealed mixed results regarding their effectiveness on increasing FA (41–44), with side effects such as increases in anxiety (45).

This systematic review aims to: 1) summarize and examine globally the available evidence regarding FA and its related individual risk factors; and 2) identify the gaps in the literature based on studies conducted worldwide. This knowledge will help both researchers and clinicians to develop more successful and well-accepted campaigns targeting specific groups in need of fertility-related information.

Specifically, this review attempts to answer two questions: 1) Are reproductive-age people informed about fertility and individual infertility risk factors? and 2) Do differences exist in FA based on gender, age, education, and reproductive status?

## Materials and methods

### Search strategy

The following electronic databases were searched from their inception through February 2017: Academic Search Complete, CINAHL plus, Education Source, ERIC, MedicLatina, MEDLINE, PsycARTICLES, PsycCRITIQUES, Psychology and Behavioural Sciences Collection, PsycINFO, Web of Science, Scielo, and Scopus. The key search terms used were: (awareness OR knowledge OR perception OR health knowledge, attitudes, practice OR fertility awareness OR fertility knowledge) AND (fertility OR fertile period OR trying ‘to’ conceive OR assisted reprod* OR delayed childbearing) and NOT (fertility awareness method* OR cancer OR HIV OR polycystic ovary syndrome OR cyst* OR botany OR zoology OR soil* OR animals OR plant* OR contracep* OR birth control) NOT (qualitative). We also included articles using a snowball sampling strategy in which the reference lists of identified articles were searched. To be eligible for inclusion, the studies had to be published in English, Portuguese, Spanish, or French. Studies published only as abstracts, dissertations, or case reports were not considered. The current review protocol is registered at PROSPERO (registration number CRD42016050186; http://www.crd.york.ac.uk/PROSPERO).

### Study screening, selection, and data extraction

**Figure 1.**
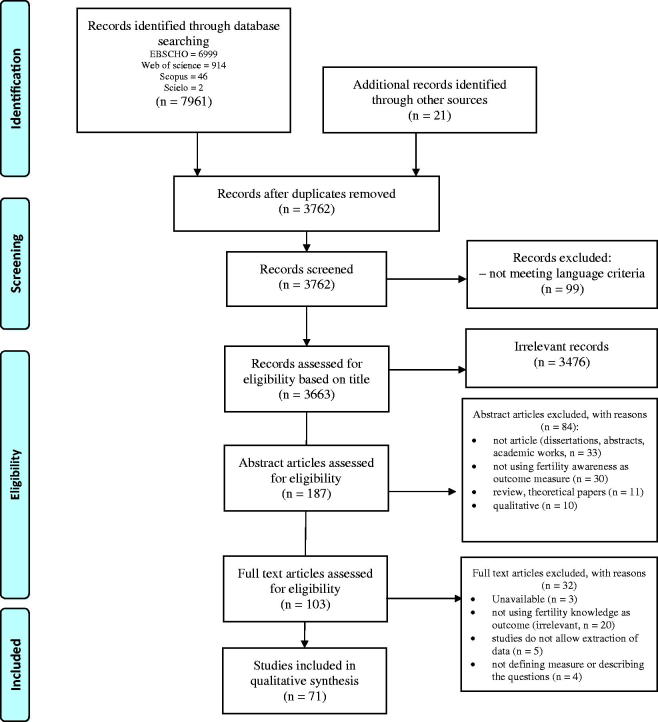
PRISMA flow diagram. From Moher et al., 2009 (46).

We followed the Preferred Reporting Items for Systematic Reviews and Meta-Analyses (PRISMA) guidelines for reporting the data analysed (46). All records were stored in a database using Endnote X6. Manual inspection was independently performed by the first and second authors, and disagreements at each stage of screening and selection were resolved by a third reviewer. The initial search identified 7961 studies. Additionally, 21 studies were found through other sources (Figure 1). During the initial screening, 3476 articles/studies were excluded based on the title and after 84 were excluded based on the abstract. Afterwards, full-text articles (*n* = 103) were independently examined and were included if they met the following criteria: 1) used quantitative data regarding FA (e.g. awareness concerning the fertile period, definition of infertility, factors affecting fertility and lifestyle risk factors, chances of pregnancy, age-related fertility decline, the success rates of medically assisted reproduction treatments [MAR], including all *in vitro* [e.g. *in vitro* fertilization, IVF] and *in vivo* treatment methods [e.g. intra-uterine insemination, IUI], and the risks of postponing pregnancy and infertility treatments); 2) used a FA-specific measure or provided a detailed description of the questions assessed. The first and second authors independently examined the included studies, and the first author extracted the relevant data, which was cross-checked by the second author. The data extracted included the author, year, and country of publication, sample size, sample characteristics, outcome measures, instruments used, and main results. To meet the second goal of this review, we recorded the associations between FA and age, gender, education, and reproductive status. A narrative synthesis approach was used to conduct this review (47).

## Results

### Study characteristics

Seventy-one studies were included in this review. Supplementary Table A1 (available online) summarizes the participant characteristics and the main findings. The studies were published between 1994 and 2017. The data originated from 26 countries, and most studies were conducted in Europe (*n* = 27), 16 in America, 12 in Asia, seven in Oceania and four in Africa. Five articles used data from more than one country. The sample sizes ranged from 20 to 7036 individuals. The majority of the studies (*n* = 41) included both men and women of reproductive age and focused on one of three populations: university/college/secondary education students (*n* = 28); people trying to conceive, those seeking fertility treatment, or both (*n* = 11); or gynaecology patients (*n* = 9). The remaining 22 studies used convenience samples. Participants were primarily in their early 20s and 30s, except those in five studies that assessed adolescents (48–52). The majority of studies were cross-sectional (*n* = 64). Five studies were pre/post-test intervention studies (41–43,45,53), and two were longitudinal studies (54,55). The baseline or first moment of evaluation data were extracted from the intervention and longitudinal studies and included in this review. Of the 71 studies included, 49 investigated the relationship between FA and variables such as age, education, gender, and reproductive status. Although all of the studies focused on FA, they varied in the way that this awareness was measured. FA was measured using specific self-report questionnaires or interviews. Supplementary Table A1 (available online) gives the measures used by each study. A minority of studies provided only a total FA score (*n* = 8), with the remaining studies reporting the results for each item separately. The included quantitative questionnaires used different response scales and formats composed of true/false, multiple-choice, or open-ended questions. Only 11 studies presented data regarding the psychometric properties of the used instruments, with the majority reporting Cronbach’s alpha of >0.70 or ‘satisfactory’ reliability (30,37,45,56–62), and one reporting a 0.52 reliability (63). The response rate ranged from 15% to 100%, but this value was only reported by 63% (*n* = 37) of the 59 studies that could have provided a response rate.

### Quality assessment

Because the answer to our second research question requires statistical inference, we performed quality assessment on the studies investigating the relationship between FA and the other variables (*n* = 49). Using a standardized quality framework for non-intervention studies (64) used for previous reviews (65,66), two researchers independently evaluated the studies. Most of the studies were rated as high quality (scores >4), and only one was rated as having low quality (67). Numerous studies failed to garner a positive score regarding the study methodology because detailed information indicating the measures used or the procedures/instruments developed, adapted, or validated was missing.

### Are people informed about fertility?

The topics explored included awareness regarding the definition of infertility, the factors affecting fertility and lifestyle risk factors, age-related fertility decline, the fertile period, and the consequences of delaying childbearing. The majority of studies explored awareness regarding more than one of these topics. To answer our first question, the findings were organized into FA topics, and then each FA topic was rated based on the described mean, standard deviation, and range or percentage of correct answers, where FA was considered low when <40%, moderate when 40%–59%, and high when ≥60% (68). The results are summarized in Supplementary Table A1 (available online) and detailed in the Supplementary data (Supplementary Tables B1 to B6, available online).

### Overall fertility awareness

Eight studies considered FA as a single construct and reported total scores (see Supplementary Table B1, available online). Four found moderate levels of awareness (45,57,59,60), three found low levels of FA (43,53,69), and the remaining study (54) found high awareness regarding female body anatomy (M = 86.4%; SD =12.7) and assisted reproductive technology (ART; includes *in vitro* treatments such as IVF treatment) (M = 62.9%; SD =20).

### Specific dimensions of fertility awareness

*Infertility definition.* Seven of nine studies assessed awareness concerning the definition of infertility (Supplementary Table B2, available online) and found that, on average, fewer than half of the people knew the definition of infertility, ranging from 14.4% (70) to 50% (71). Two studies found that people were familiar with the term ‘infertility’ (50,72).

*Age-related fertility decline.* A detailed description of the findings regarding awareness of the age-related fertility decline is presented in Supplementary Table B3 (available online). Nine of 12 studies found moderate-to-high awareness (42% to 93%) (30,35,42,63,67,73–76), and three found low awareness (49,77,78) regarding age as risk factor.

Of the 16 studies that analysed awareness regarding women’s most fertile age, eight found high awareness (33,36,79–84), six reported moderate awareness (between 38% and 52%) (37,62,78,85–87), and two observed low awareness (41,88).

A total of 37 studies explored awareness regarding the age-related fertility decline and the chances of becoming pregnant, both spontaneously and through MAR. The majority found low levels of awareness (*n* = 23). In general, people were aware of the decline in fertility due to age because they estimated a lower likelihood of becoming pregnant at older ages (89,90). However, they also seriously overestimated the fertility potential and the chances of becoming pregnant, both spontaneously and through MAR treatment (33–37,41,61,62,71,76,78,80–82,84–98). Six studies found that a high percentage of participants believed that MAR treatments compensate for the age-related fertility decline (42,58,63,73,93,99). One exception to this pattern was the awareness regarding the marked decline of fertility due to female age, with nine studies reporting that between 40% and 90% of participants provided a correct answer or an accurate estimate (34,42,58,61,63,80,81,88,97). Five other studies also found moderate-to-high awareness regarding the effect of age on treatment success and the age at which the chances of pregnancy decrease (55,73,79,99,100).

*Infertility risk factors.* Of the nine studies exploring participants’ awareness about the male and female causes of infertility (see Supplementary Table B4, available online), three found that less than 40% of the sample correctly answered this question (42,101,102). Four studies found moderate levels of awareness (58,63,70,103), and two found high levels of awareness (50,104).

The studies exploring awareness about the causes of infertility (*n* = 11) found mixed results (detailed results in appendices, see Supplementary Table B4, available online), with two studies finding low awareness (87,102), two finding high awareness (101,103), and the remaining seven finding moderate or both low and high awareness regarding different causes of infertility (30,49,70,74,75,94,97). Some studies also found that a significant percentage of participants believe in certain myths (e.g. the previous use of contraceptive pills reduces fertility) (30,58,63,70,97,103).

The 18 studies exploring awareness of sexually transmitted infections (STIs) as a risk factor for fertility found that approximately 30% (70) to more than 70% (e.g. (94)) recognized or mentioned STIs as a risk factor for infertility.

Regarding the infertility risk factors related to lifestyle (e.g. smoking, weight, and drugs), the majority (*n* = 16 of 30, see Supplementary Table B4, available online) found high awareness levels (31,33,41,42,63,73–76,80,91–93,97,101,105). Two studies found moderate FA (30,54), three reported low awareness (49,69,77). Nine studies found mixed results: four studies found both high and low awareness regarding different risk factors (48,87,94,103), two found both low and moderate awareness (78,95), one found both moderate and high awareness (58), and two found low, moderate, and high awareness for different risk factors (30,82).

*Fertile period.* Of the 11 studies assessing awareness regarding the fertile period (Supplementary Table B5), four found that a low percentage of participants were able to identify the ovulation period (52,95,106,107); three studies found moderate awareness (86,103,108), and four studies found high awareness (74,94,97,104).

*Consequences of delaying childbearing.* Thirteen studies explored awareness regarding the negative consequences of delaying pregnancy (see Supplementary Table B6, available online). Seven studies found that >50% of participants knew that women aged 35 and over are more likely to have problems becoming pregnant and are at an increased risk of medical problems during pregnancy and being diagnosed with genetic anomalies (55,56,73,76,109–111). Six studies exploring awareness regarding the increased risk of miscarriage with age found high awareness (42,58,63,73,74,76), and one found low awareness (35). A small percentage of participants knew that women aged 35 and above are more likely to have multiple births, caesarean sections, preterm infants, low-birth-weight infants, and stillbirths (56,73,111) as well as that children born to fathers >45 years old have higher rates of learning disabilities, autism, schizophrenia, and some cancers (42). Machado and colleagues (77) found that only 32% of the students assessed knew that postponing the age of childbearing is associated with a high-risk pregnancy.

### Do differences exist in FA based on gender, age, education, and reproductive status?

Supplementary Table A2 (available online) presents the results of the 49 studies examining the associations between FA and the aforementioned variables.

*Gender*. Women presented greater FA than men in 12 studies (34,48,57,60,69,80,87,95,96,98,103,110); however, a similar number of studies (*n* = 10) found no significant gender differences (31,45,49,50,55,62,82,85,88,89). Some studies also presented mixed results (33,36,37,84) (see Supplementary Table A2, available online).

*Age*. Approximately half of the studies (*n* = 11) did not find significant associations between age and FA (31,53,58,60,63,75,81,94,97,104,110); nine studies found that older people had higher FA (43,52,56,57,86,98,99,103,109); and only one found that younger participants (<30 years old) presented with higher FA (76). Two studies presented mixed results (74,106).

*Education*. Thirty studies investigated the association between education and FA. Higher education was associated with greater FA in 18 studies (31,43,52–54,56,57,63,67,80,95–97,99,104,106,109,110). Five studies did not find significant associations (48,55,58,75,86). One study found that participants with a university degree had greater FA than those with a non-university education. However, this difference was not statistically significant among participants trying to conceive (60). Higher levels of FA were found among medical/health students versus other study areas (69,80,82,89) and among gynaecologists compared with other physicians and nurses (100). Only one study did not find statistically significant differences in FA between humanities and science students (84).

### Reproductive status

Five of the eight studies focusing on fertility status found that participants with a current or previous infertility diagnosis had greater FA than those without a diagnosis (60,67,76,109,111); one study found no association (30), and two studies (55,73) found inconsistent associations (see Supplementary Table A2, available online, for detailed information). Regarding childbearing status, five of the nine studies found no significant differences between parents and non-parents (53,55,57,75,86), and two found that participants who had child(ren) had greater FA than non-parents did (54,106). Only one study found that parents had less FA that non-parents (99), and another found a mixed pattern (60).

Participants who had previously planned their pregnancy showed greater FA than those who did not in both studies exploring these differences (56,111). Two studies did not find differences between participants who experienced a pregnancy and those who did not (43,81). One study found a positive association with having experienced a pregnancy (76), but another found a negative association (109). Four other studies found that people planning to have children before the age of 30 showed similar levels of FA to those planning parenthood after 30 years in one study (36); however, the desire to have children was associated with having higher FA in another study (43). In addition, a mixed pattern was found in one study (89), but another one did not find a significant association (110).

## Discussion

This paper provides an up-to-date systematic review of the available evidence on FA worldwide. It is the first to investigate the level of FA and its associations with sociodemographic and reproductive variables in both women and men. After two researchers independently conducted rigorous selection and data extraction processes, 71 studies were included in this review.

### FA: are people informed about fertility and infertility individual risk factors?

This worldwide review suggests that FA levels were low to moderate among people of reproductive age. Although this population is familiar with the term infertility (50,72), awareness is generally low and certain myths remain. In general, people were aware that age poses a high risk for reduced fertility; however, they also believed that age-related fertility decline starts later than the actual turning point, and they overestimated the chances of becoming pregnant both spontaneously and through fertility treatment. This overestimation might have been reinforced by schools’ sexual education curricula, which are generally focused on preventing pregnancy (112).

Awareness regarding the remaining risk factors was generally high. Two exceptions were two studies from Nigeria and Ukraine, which found low awareness regarding STIs. These results might be related to cultural and content differences regarding sexual education curricula. This difference is particularly relevant because STIs are responsible for a higher proportion of fertility problems in developing countries, both in women and men (70). The high awareness levels regarding the risk factors related to lifestyle might be explained given that smoking, alcohol, and drugs are common risk factors for other well-known chronic diseases (e.g. cardiac disease and cancer). The high awareness of female risk factors compared with male risk factors can be specific of reproductive health which might be a reflection of the higher focus on women’s health in the reproductive and sexual health-care services (113). However, awareness regarding the impact of increasing female age on preterm delivery, low birth weight, and caesarean section was low (55,56,111), as was awareness regarding reproductive outcomes on the offspring (autism, schizophrenia, learning disabilities) due to men’s advanced age (42,58).

### Variables associated with FA

The findings of this review suggest that women have greater FA than men (12 studies), which might be expected because men are less engaged in preconception planning and counselling (113–115). However, 10 studies did not find significant differences between men and women, and four found mixed results (55,62,82,89). The lack of an association between gender and FA was mainly pointed out by studies using university student samples. Some studies found that women were more aware of the marked age-related fertility decrease (33,36,37), whereas men were more aware about the estimation of the number of involuntarily childless couples (33,36). Women and men students might be equally not aware of fertility issues. Studies showed that young people want to first complete their education, find a stable partner, and climb the career ladder before childbirth (36,37,62), which can result in less interest in FA. In addition, the majority of these studies have significantly fewer men than women in their samples, which might have resulted in some bias.

This review found that older people had higher FA or did not find significant associations, which might be explained by the heterogeneity of the recruited samples (e.g. (74)). Although study participants still overestimate the fertility potential, the oldest participants reported that fertility declines with age with greater accuracy. This is expected as people closer to the ‘reproductive limit’ could be more motivated to seek information and as a result of that be more aware about fertility issues.

Our findings suggested that education is positively associated with FA. People with higher education might seek more information, resulting in higher awareness, which is consistent with the available evidence regarding the association between education and fertility treatment seeking. Even in countries where fertility treatments are available in the public health system or are covered by insurance, people with higher education seek fertility treatment more often compared to people with lower education (116–118). Interestingly, education was also associated with a higher probability of being childless at the age of 30 (120), posing the question of whether awareness is enough to prevent the delay of childbearing and seeking treatment. Hence, it seems that people with more education, who are expected to be more focused on their career goals, are those who are more optimistic about being able to have the children they desire without infertility problems, even when they delay childbearing (119). This finding leads us to question the role that education plays in reproductive planning. Because people delay childbearing for various reasons (6) and the decision to have children is multifaceted (2,3), it is likely that FA only has a modest effect on reproductive decisions among highly educated people. A tendency towards higher levels of FA was found amongst patients who had undergone MAR treatments, had difficulties in trying to conceive, and in those with longer infertility. These results were expected because people facing difficulties in trying to conceive seek information about fertility and strategies to increase their chances of becoming pregnant (120). However, some studies found a mixed pattern. For example, one study found that infertile women had a higher belief that ART can overcome the effects of age than did pregnant women (73). Thirty per cent of these participants were prepared to consider the use of IVF, even if the predicted success rate for them was less than 10% for one cycle. This finding suggests that people who face difficulties with becoming pregnant accept fertility treatments regardless of their success rate, as this might be the only way to achieve childbirth.

Findings regarding pregnancy history were mixed. People who had planned their pregnancies showed greater FA than those who had unplanned pregnancies. Parents and childless people presented similar FA, assuming that 50%–79% of parents conceive spontaneously and within a one-year period (36). It seems safe to assume that these couples had no difficulties in becoming pregnant (81) and have similar FA levels as those who have not started trying to conceive (e.g. (36)).

### Strengths, limitations, and recommendations for future research

This paper has strengths that should be highlighted. The *a priori* review protocol was designed following PRISMA guidelines and registered at PROSPERO. The study search was performed using 12 databases from their inception date to search for and obtain the best published evidence concerning FA. Moreover, two independent researchers performed the study selection and quality assessment, leading to a more rigorous assessment of the findings and higher confidence in the results regarding the factors associated with FA. Because we included studies from five continents and these data originated from very different populations, we obtained a broader and more global view of the topic. Although a significant proportion of studies exclusively focused on women (24 of the 71 studies), the majority also included men, who are often thought of as underrepresented in infertility research (66).

However, certain limitations should be noted. First, the heterogeneity and diversity of the samples studied and the different sample sizes create challenges when summarizing the findings. Although we attempted to explore the associations among the different population samples to understand their relationship to FA levels, not every study explored these relationships. Second, we believe that certain discrepancies within our findings might reflect the different ways of assessing FA because the findings were based both on studies using structured instruments and on those using a set of questions classified as correct/incorrect; only a small proportion applied psychometrically validated instruments. Research focusing on FA should better define their instruments and examine their psychometric properties as claimed by some authors (121,122). In addition, the use of different analytic strategies (e.g. univariate or multivariate analyses or using age as a continuous variable or age groups as a categorical variable) and the lack of subgroup analyses, especially those regarding age and reproductive status, might also have contributed to the current mixed findings. Third, most of the samples were from the USA or Europe, which increases the likelihood of a cultural bias.

The findings of this review indicate the presence of three major gaps in this field that should be addressed in future studies: 1) the lack of validated and reliable FA instruments; 2) the scarce differential findings that do not allow a clear picture of who is in need of this awareness and during what phases within the reproductive life plan this knowledge should be disseminated; and 3) the lack of evidence regarding the best ways to disseminate this information. Studies with long-term follow-up assessments and randomized controlled trials are necessary to better understand the role played by FA in preconception care, reproduction, and parental projects.

### Implications for clinicians and policymakers

The current available evidence offers clues about how different populations might require specific, targeted knowledge. These implications are particularly relevant because delivering non-personalized information seems too often to result in a public opinion of people being pressured not to postpone childbearing, ignoring well-known factors that influence this decision such as career goals, stable partner, finances, etc. These top-down strategies, mainly developed by governments and focused on a simple message, were found to have limited success in improving the adoption of health behaviours (123,124).

Because men play an important role in reproductive health and childbearing decisions (125) and our findings suggest lower FA levels in men, it might be important to rethink reproductive health to be more male inclusive, including FA campaigns and preconception care. Having a reproductive plan results in better reproductive outcomes, as shown by our findings of higher FA among individuals who have planned their pregnancies than those who had unplanned pregnancies. While preconception care programmes are available in the majority of developed countries, there is no consensus about the content (126,127) and the target population that may benefit more from it (e.g. reproductive age, planning a pregnancy, high-risk of reproductive outcomes). In this sense, preconception programmes should provide personalized counselling in order to meet individual (both male and female) or couple reproductive goals. For example, the Reproductive Life Plan (128) is a programme directed to people of reproductive age that can be used by health-care professionals in their routine practice to counsel in a tactful way the men and women and guide them to find strategies in order to achieve their reproductive goals (129).

In addition, health-care services and sexual education programmes should be more inclusive of adolescents with low socioeconomic status and young adults with low education, given the findings that suggest these groups have low FA. People from developed countries (4) and engaged in higher education (130) might benefit more from these interventions, since they tend to postpone childbearing. Recently, social egg freezing has been offered as an option to postpone childbearing. However, studies have shown that people tend to seek egg freezing at a later age (131); thus, young people should be counselled about the optimal age to do it (between 25 and 34 years old) and should be informed about success rates and risks involved (132).

On the other hand, infertile couples might benefit more from interventions about the effectiveness of fertility treatments, given their low awareness regarding this aspect. Information concerning realistic success rates might prevent an increase in treatment burden, reduce the negative emotional effects of high expectations, and contribute to a better quality of life during treatment (133).

Health authorities can work together with schools, health-care professionals, and patient associations to develop these interventions, where young adolescents/young adults and patients can be listened to and have a more active role guided by professionals. These interventions should follow a patient-centred perspective, which has been linked to greater effectiveness and satisfaction (134). Additionally, the way of dissemination of awareness is also important, and further research is needed to find the best format to adopt. While written information (e.g. leaflets) is associated with positive outcomes in increasing health knowledge (44), its effect on actual health behaviour is not known. There is evidence that web-based educational interventions positively impact health behaviour (135). In the field of reproductive health, online websites seem to be a good resource of information in preconception and fertility education (42,136).

Previous interventions effectively increased FA over short-term periods (e.g. 41,43,44); however, FA decreased six months afterwards (42). It might be important to examine the effectiveness of tailored interventions that might benefit those interested in gaining FA by incorporating well-known factors that influence fertility decisions (e.g. career goals, meaningful relationships, or financial stability).

## Conclusions

Although we found an increase of studies focusing on fertility awareness (FA), the quality and consistency of their findings are lacking. The findings of this review suggest that FA among reproductive-age people is low to moderate. The evidence suggests that women, more educated people, people having difficulties with conceiving, and those who planned their pregnancies have greater FA levels. Having or desiring to have children was not related to FA levels. Age was inconsistently associated with FA, with some studies indicating that higher age was associated with FA but others showing the opposite or no association with FA.

## Supplementary Material

Supplemental Material

Supplemental Material
