# Supplementary material for: What do people know about fertility? A systematic review on fertility awareness and its associated factors
Source: Ups J Med Sci. 2018 Jun 29;123(2):71–81. doi: 10.1080/03009734.2018.1480186 (PMC6055749; doi:10.1080/03009734.2018.1480186)
Supplement: Supplemental Material [file IUPS_A_1480186_SM6860.zip › Pedro_supp_matt/Supplemental_Data_B.docx]

**What Do People Know about Fertility? A Systematic Review on Fertility Awareness and its Associated Factors**

**Juliana Pedro*, Tânia Brandão, Lone Schmidt, Maria E. Costa and Mariana V. Martins**

**Supplemental Data B**

| **Table B.1. Studies assessing fertility awareness based on total score.** | | | | | |
| --- | --- | --- | --- | --- | --- |
| **Authors, year** | **Instrument** | **Sample** | **Low (<40%)** | **Average (40-59%)** | **High (≥60%)** |
| Bunting et al., 2013 | Cardiff Fertility Knowledge Scale (CFKS)(13true/false questions) – Bunting et al., 2013 | People trying to get pregnant |  | 59.09%(SD=24.7) |  |
| Childress et al., 2015 | Fert-AP survey (13 questions on female reproductive anatomy,  ART, and fertility factor awareness knowledge) | Women attending first infertility visit |  |  | female body knowledge: M=86.4%; SD= 12.7%;  infertility/ART knowledge: M= 62.9%; SD= 20%; |
| Fugener et al., 2013 | Knowledge concerning reproduction and fertility (8 questions)- Fugener et al., 2013 | Students | M reproduction knowledge =6.3 (range 0-16) |  |  |
| Fulford et al., 2013 | Cardiff Fertility Knowledge Scale (CFKS)(13true/false questions) – Bunting et al., 2013 | Childless women |  | M=51.09% |  |
| Garcia et al., 2016 | Fertility knowledge Questionnaire (10 multiple-choice questions) – Garcia et al., 2016 | Women candidate to oocyte donation | M=3.7 (SD=1.6)  Range 0-10 |  |  |
| Maeda et al., 2015 | Cardiff Fertility Knowledge Scale (CFKS)(13true/false questions) – Bunting et al., 2013 | Group trying to get pregnant; group from general population |  | Triers group: 53.1 (SD=23.4);  General group: 44.4 (SD=23.1) |  |
| Maeda et al., 2016 | Cardiff Fertility Knowledge Scale (CFKS)(13true/false questions) – Bunting et al., 2013 | General population desiring to have children |  | M women=49.5 (SD=23.9);  M men=42.5 (SD=24) |  |
| Stern et al., 2013 | Knowledge about different aspects of reproduction (6 item questionnaire)-Stern et al., 2013 | Women seeking contraceptive counselling | M=6.2(range 0-20) |  |  |
| Note: Considering the range scores, the means and percentage of correct responses were classified in low, average or high based on less than 40%, between 40 and 60% and more than 60% correct answers/median answers of the sample. | | | | | |

| **Table B.2. Studies assessing awareness regarding infertility definition (based on percentage of people that give a correct definition of infertility).** | | | | |
| --- | --- | --- | --- | --- |
| **Authors, year** | **Sample** | **Low (<40%)** | **Average (40-59%)** | **High (≥60%)** |
| Adashi et al., 2000 | General population |  | ≈50% |  |
| Ali et al., 2000 | General population | 25% |  |  |
| Al Khazrajy et al, 2009 | Patients seeking fertility treatment |  | 50.7% |  |
| Bennett et al., 2015 | Patients seeking fertility treatment |  | 49% |  |
| Conceição et al., 2017 | Students |  | M=2.83 (SD=1.02; range 0-5) |  |
| Ikimalo & Babatunde, 2012 | General population | 32% |  |  |
| Iliyasu et al., 2013 | General population | Women: 14.4%, Men: 21.7% |  |  |
| Quach and Librach, 2008* | Students |  |  | Women: 82.18%, Men:77.1% |
| Sugiura-Ogasawara et al., 2010* | General population |  |  | 98.8% |
| *note: these studies reported the percentage of people “familiar” with term infertility. | | | | |

| **Table B.3. Studies assessing age as fertility risk factor and age-related fertility decline (based on percentage of correct answers).** | | | | |
| --- | --- | --- | --- | --- |
| **Authors, year** | **Sample** | **Low (<40%)** | **Average (40-59%)** | **High (≥60%)** |
| Age as risk factor for fertility (n=12) | | | | |
| Abolfotouh, et al., 2013 | Patients seeking fertility treatment |  | marriage at advanced age: IVF patients: 53.8%, outpatients: 43.5% |  |
| Al Khazrajy et al., 2009 | Patients seeking fertility treatment |  | 51.3% knows age affect IUI success  59.6% knows age affect IVF success |  |
| Bretherick et al., 2014 | Students |  | 46% |  |
| Daniluk & Koert, 2015 | General population |  | Men age: 52.8% |  |
| Daniluk et al., 2012 | Women (general population) |  | Men age: 42.8% |  |
| Daumler et al., 2012 | Men (general population) |  |  | Men age (older than 45 years): 69.5% |
| Deatsman et al., 2016 | Women seeking gynaecologic/obstetric care |  |  | 79% |
| Lundsberg et al., 2014 | Women |  |  | 85 % (mean of 2 items) |
| Machado et al., 2014 | Students | 18.7% |  |  |
| Maheshwari et al., 2008 | Subfertile and pregnant women |  |  | 93% subfertile, 88% pregnant |
| Pitts & Hanley, 2004 | Students | Age: 33.6% |  |  |
| Tyden et al., 2006 | Students | 17% |  |  |
| Most fertile woman´age (percentage of correct answers or mean age indicated by participants, n=16). Correct answer based on Lampic et al., 2006: 20-24 years old. | | | | |
| Abiodun et al., 2016 | Students |  | 41.1% |  |
| Chan et al., 2015 | Students | 16% women, 27% men |  |  |
| Conceição et al., 2017 | Students | Range M age answered =18 to 32 |  |  |
| Ekelin et al., 2012 | Students |  |  | M women=22  M men = 22 |
| Garcia et al. 2015 | Women candidate to oocyte donation |  | 51.5% |  |
| Hodes-Wertz et al.,2013 | Women completed >1 cycle of cryopreservation |  |  | 95% |
| Lampic et al., 2006 | Students |  | 46% men | 63% women |
| Meissner et al., 2016 | Students |  |  | 77% |
| Mogilevkina et al., 2016 | Medical students |  | 47% women, 43% men |  |
| Mortensen et al.,2012 | Women |  |  | 77% |
| Nouri et al., 2014 | Students (medical and non-medical) |  |  | Between 70 and 89% |
| Petersen et al., 2015 | Women in fertility clinic |  |  | 72% |
| Peterson et al., 2012 | Students |  | 44% women, 38% men |  |
| Rovei et al., 2010 | Students |  |  | 69% women, 60% men |
| Sorensen et al., 2016 | Students |  | 50% men | 61% women |
| Tyden et al., 2006 | Students |  | 51% |  |
| Age of fertility decline, chances of becoming pregnant spontaneously and through MAR treatments (percentage of correct answers or mean of ages indicated by participants, n=37  Reference for considering correct answers (based on Lampic et al., 2006): Slight decline: 25-29 years; Marked decrease: 35-39%; Chance of becoming pregnant during ovulation: 30-39%; Chance of becoming pregnant spontaneously: 70-79% if women is 25-30 years; 50-59% if women is 35-40 years; IVF success rate :20-29%. | | | | |
| Abiodun et al., 2016 | Students | Slight decline: 4.1%  marked decrease: 6.2%  Chance of becoming pregnant during ovulation: 0.8%  Chance of becoming pregnant if women is 25-30: 19%  Chance of becoming pregnant if women is 35-40: 14.1%  Chance of becoming pregnant thought IVF: 6.4% |  |  |
| Adashi et al., 2000 | General population | success with IVF: 22% |  |  |
| Bavan et al., 2011 | Students | Age of fertility decline: 38% |  | Woman is born with all eggs: 89%  IVF allows a woman to conceive with reduced egg supply: 40% saying false; 60% correct answers |
| Bretherick et al., 2014 | Students | Best chance of becoming pregnant at the age of 40:egg donation:15.2%  Change of becoming pregnant at 20, 30, 40: overestimate |  | age of fertility decline: 70.3% |
| Chan et al., 2015 | Students | Slight decline:6% women,9% men  Marked decrease: 37% men  Chance of becoming pregnant spontaneously and by IVF: <21%  Chance of becoming pregnant during ovulation: 5% women, 9% men | Marked decrease: 48% women |  |
| Chelli et al., 2015 | Midwifery students | Slight decline:22.5%  Chance of a 35 years old woman get pregnant in a month: 26% |  |  |
| Conceição et al., 2017 | Students | Slight decline: M= 35.98; SD=5.5  Marked decrease: M=42.03; SD=5.24 Chance of becoming pregnant during ovulation: M=83.77; SD=19.22  Age of decrease in success of IVF: M=40.96; SD=5.17  MAR success if woman is 35 years: M=58.46; SD=18.07  MAR success if woman is 35 years: M=46.32; SD=29.12  MAR success if woman is 35 years: M=32.87; SD=20.74 | Chance of becoming pregnant if woman is 25-30 years: M=83.78; SD=13.68  Chance of becoming pregnant if woman is 35-40 years: M=66.12; SD=17.30 |  |
| Daniluk & Koert, 2013 | Men (general population) | ART treatments can overcome the effect of age: 7.9% | Fertility decline in men at 50: 42.4%  Need for more than 1 cycle to have a baby: 46.2% | Fertility decline in women at 35 years: 71.2% |
| Daniluk & Koert, 2015 | General population | ART treatments can overcome the effect of age: 4.5% | Fertility decline in men at 45: 40.7% | Marked decrease: 80.9% ;  Need for more than 1 cycle to have a baby: 60.3% |
| Daniluk et al., 2012 | Women (general population) | ART treatments can overcome the effect of age: 9.1% |  | Fertility decline in women at 35 years: 90.3%  Need for more than 1 cycle to have a baby: 66.1% |
| Deatsman et al., 2016 | Women attending gynaecologic/obstetric care | Fertility decline at 35 years: 30.5% |  |  |
| Ekelin et al., 2012 | Students | Slight decline, marked decrease: overestimate (ages 37; 44-47)  IVF success: overestimate (45-49chance of success)  Chance of becoming pregnant during ovulation: overestimate (chance 64.5-72.5) |  |  |
| Fotopolou et al., 2015 | Medical students | Slight decline: 15.9% |  |  |
| Garcia et al., 2017 | Health professionals | Age limit to spontaneous pregnancy: 39% |  | Age limit to pregnancy thought IVF: 78.5%  Age limit for a pregnancy with oocyte donation: 82.5% |
| Garcia et al., 2015 | Women candidate to oocyte donation | Fertility decline: 26% |  |  |
| Guedes et al., 2014 | General population (pregnant couples) |  | Fertility decline: women: 55.80%; men: 51.60% |  |
| Hammarberg et al., 2016 | Nurses | Female Fertility decline: 34%  Male fertility decline: 18%  IVF success if women is 35 years: 15%  IVF success if women is 40-44 years: 29% |  |  |
| Hammarberg et al., 2013 | Women | Female Fertility decline: 31% women, 20% men  Male fertility decline: 31% women, 36% men |  |  |
| Hashiloni-Dolev et al., 2011 | Students | Chance of becoming pregnant spontaneously (overestimated for all age stages)  Chance of becoming pregnant thought IVF (overestimated for 40-43, 44-47, 48-52, 53-58 age stages) |  | Success of IVF in 20-35 years, 36-39 years (accurate estimates) |
| Hodes-Wertz et al.,2013 | Women completed >1 cycle of cryopreservation |  | 43% |  |
| Holton et al., 2016 | Men (general population) | Age of woman fertility decline: 31.3% | Age of men fertility decline: 45.5%  ART can overcome the effect of age (percentage answering No, I don’t know): 40.5% |  |
| Lampic et al., 2006 | Students | Slight decline, marked decrease: <37%  Chance of becoming pregnant during ovulation: <8%  Chance of becoming pregnant if women is 25-30, 35-40 years and thought IVF: <24% |  |  |
| Lucas et al., 2015 | Students | Chance of becoming pregnant spontaneously (overestimated for 20-25, 26-30, 31-35, 41-45, 46-50)  Chance of becoming pregnant thought IVF (overestimated for 40-43, 44-47, 48-52, 53-58 age stages) |  | Chance of becoming pregnant spontaneously for age stage 36-40 years (accurate estimate)  Chance of becoming pregnant thought IVF for age stage 20-35 years (accurate estimate) |
| Maheshwari et al., 2008 | Subfertile and pregnant women | ART can overcome the effect of age (percentage answering No): 15% subfertile; 23% pregnant | Effect of age no IVF success: 53% subfertile, 46% pregnant | IVF success: 75% subfertile, 69% pregnant |
| Meissner et al., 2016 | Students | Slight decline: 36%  Chance of pregnancy during ovulation: 14%  IVF success: 32% | Marked decrease: 46% |  |
| Mogilevkina et al., 2016 | Medical students | Slight decline: 9% women, 10% men  Marked decrease: 11% women, 9% men  Chance of pregnancy during ovulation: 2% women, 4% men  Chance of pregnancy spontaneously: <22%  IVF success: 11% women, 10% men |  |  |
| Mortensen et al.,2012 | Women | Slight decline: 33%  Chance of becoming pregnant spontaneously during ovulation, at 25-30 and 35-40 years: <29%  IVF success at age 25-30 and 40-43: <34% | Marked decrease: 57% |  |
| Nouri et al., 2014 | Students (medical and non-medical) | Marked female decrease: 13-18%  Slight male decrease: 18-26%  Marked male decrease: 14-26% | Slight female decrease: 42-62% |  |
| Peterson et al., 2012 | Students | Slight decrease: 8-18%  Marked decrease: 14-24%  Chance of pregnancy during ovulation: 2-3%  Chance of pregnancy spontaneously: 14-19%  IVF success:10-11% |  |  |
| Rovei et al., 2010 | Students | Slight decrease: 7-10%  Marked decrease: 9-11%  Chance of pregnancy during ovulation: 13-21%  IVF success:18-28% |  |  |
| Sorensen et al., 2016 | Students | Slight decrease: 30% men  Marked decrease: 31% women; 29% men  Chance of pregnancy during ovulation: 7% women; 11% men  Chance of becoming pregnant if women is:  - 25-30 years: 18% women; 29% men  -35-40 years: 14% women; 11% men  IVF success:25% women; 29% men | Slight decrease: 45% women |  |
| Svanberg et al., 2006 | Students | Marked decrease: 35% women  Chance of pregnancy spontaneously: 21-28%  IVF success:22-23% | Marked decrease: 48% women |  |
| Stoebel-Ritchter et al., 2012 | General population | Fertility decline: 3%  MAR success: 21-22% |  |  |
| Swift & Liu, 2014 | Women attending a fertility clinic | Percentage of couple experiencing infertility: 17.1%  IVF success when women is 30 years old: 26.4%  IVF success when women is 40 years old: 35% | Marked decline: 54.3% |  |
| Tyden et al., 2006 | Students | Slight decline: 10%  Marked decrease: 6%  Chance of becoming pregnant during ovulation: 4%  Chance of becoming pregnant if women is 25-30, 35-40 years and thought IVF: 33%; 25%; and 23% respectively |  |  |
| Vassard et al., 2016 | General population | Marked decrease: 20% men  Chances of becoming pregnant spontaneously: 31-34% |  | Slight decrease: 73-83% Marked decrease: 60% women |
| Virtala et al., 2011 | Students | Slight decrease: 14% women, 9% men  Marked decrease: 22% women, 11% men  Chances of becoming pregnant spontaneously: 15-29% |  |  |

| **Table B.4. Studies assessing awareness regarding factors affecting fertility/risk factors and causes of infertility.** | | | | |
| --- | --- | --- | --- | --- |
| **Authors, year** | **Sample** | **Low (<40%)** | **Average (40-59%)** | **High (≥60%)** |
| Recognized both male and female equally cause of infertility (n=9) | | | | |
| Ali et al., 2011 | General population |  | 40% |  |
| Bennett et al., 2015 | Patients seeking fertility treatment |  |  | 94% |
| Daniluk et al., 2012 | Women (general population) |  | M=2.6 (SD=0.92), range 0-5 |  |
| Daniluk & Koert, 2013 | Men (general population) |  | M=2.9 (SD=0.90), range 0-5 |  |
| Daniluk & Koert, 2015 | General population | 38.7% |  |  |
| Ikimalo & Babatunde, 2012 | General population | 21.3% |  |  |
| Iliyasu et al., 2013 | General population |  | 50.8% |  |
| Ola et al., 2010 | General population | 8% |  |  |
| Quach & Librach, 2008 | Students |  |  | 78.4% |
| Causes of infertility (n=11) | | | | |
| Abolfotouh, et al., 2013 | IVF patients and outpatients |  | Abnormal menses: outpatients: 43.1%  Infections of genitourinary tract in women: IVF: 50%, outpatients: 61%; in men: IVF: 55.8%, outpatients: 62.1% | Abnormal menses: IVF patients; 64.4%; Blocked tubes: IVF patients: 77.8%; outpatients: 65.8%; |
| Ali et al., 2011 | General population |  |  | Abnormal menses: 85% , blocked tubes: 94%, infections on female and male genitourinary tract: 72 and 74% |
| Daumler et al., 2016 | Men of reproductive age | Hernia repair: 24.4%  Delayed puberty: 22.1%  Size of testicles: 19.15% | X-rays: 46.7% | Cancer treatment such as radiation or  Chemotherapy: 76.6%  Genetic abnormality: 69.8% |
| Hammarberg, 2016 | Nurses |  | Irregular cycles: 52% |  |
| Ikimalo & Babatunde, 2012 | General population |  |  | Risky sexual behaviour: 100%  Abnormalities of reproductive organs:93.3%  Medical diseases: 93%  Previous abortion: 88.7% |
| Iliyasu et al., 2013 | General population | Blocked tubes:28%  Menstrual irregularities: 25%  Induced abortions:32% | Pelvic infections: 55.2% |  |
| Lundsberg et al., 2014 | General population | Painful periods: 30.4% |  | Irregular peridos:72.7% |
| Mogilevkina et al., 2016 | Medical Students | Genetic disorders affect female fertility: 12% female, 6% male  Abortion affect female fertility: 16% female, 9% male  Genetic disorders affect male fertility:9% female, 6% male |  |  |
| Ola et al., 2010 | General population | Diseases in men and women:8%  Abortions:31.8% |  |  |
| Pitts & Hanley, 2004 | Students | Influence of genes: 17.8% | Problems with ovulation: 40.3%  Problems with sperm count: 57.9% |  |
| Swift & Liu, 2014 | Women attending fertility clinic | Ovarian cysts: 5.7% | Pelvic inflammatory disease: 57.1% | Irregular menstrual cycles: 74.3%  Endometriosis: 62.1% |
| Misconceptions and myths (*percentage of people believing in these myths, n=11) | | | | |
| Abolfotouh, et al., 2013 | IVF patients and outpatients | IVF patients, Outpatients  black magic: 72.1%, 65.8%*  Djinns/supernatural causes: 63.4%, 56.1%  Vigorous exercise: 20.2%, 19.3%  previous use of contraceptive pills: 44.3%, 42.4%  previous use of intrauterine devices: 78.8%, 68.4% | | |
| Ali et al., 2011 | General population | Previous use of contraceptive pills: 61%*  Previous use of intrauterine devices: 53%*  Supernatural causes:30%*  Black magic:38%* | | |
| Bavan et al., 2011 | Students | oral contraceptives preserve healthy egg supply: 20%**  regular menstrual cycles with oral contraceptives assure a healthy supply: 19% **  Exercise and diet as preserve egg supply: 78%** | | |
| Bunting & Boivin, 2008 | Students | 58% believed in myths* (living in the countryside; eating 5 portions of fruit a day)  73% believed healthy habits could increase chance of pregnancy: 73%* (e.g., less than 7 minutes of exercise per day) | | |
| Daniluk & Koert, 2013 | Men (General population) | Myths: Previous use of contraceptive pills: 42%**  Fitness level and health is better indication of fertility than age: 57.3%** | | |
| Daniluk & Koert, 2015 | General population | Previous use of contraceptive pills: 40.2% *  Fitness level and health is better indication of fertility than age: 65.3%* | | |
| Daniluk et al., 2012 | Women (General population) | Myths: Previous use of contraceptive pills: 32.7%**  Fitness level and health is better indication of fertility than age: 57.2%** | | |
| Ikimalo & Babatunde, 2012 | General population | Punishment from God: 1.4%*  Curse from witchcraft/enemies: 4.7%** | | |
| Iliyasu et al., 2013 | General population | Evil spirit: 92.1%*  Previous use of contraceptive pills:7%*  Previous use of intrauterine devices:5%*  God´s will: 4% | | |
| Ola et al., 2010 | Women attending fertility clinic | Supernatural causes:26,5%* |  |  |
| Swift & Liu, 2014 | Women attending fertility clinic | Myths: **  -previous/prolonged use of contraceptive pills: 42.9% **  -vegetarian diet:5.7%**  - alcohol: 1 drink/week: 20.7%** |  |  |
| Recognized/mentioned sexually transmitted infections as risk factor (n=18) | | | | |
| Al Khazrajy et al, 2009 | Patients seeking fertility treatment |  |  | 75% |
| Daniluk & Koert, 2013 | Men (general population) |  |  | 63.2% |
| Daniluk & Koert, 2015 | General population |  |  | 73.4% |
| Daniluk et al., 2012 | Women (general population) |  |  | 82.2% |
| Daumler et al., 2016 | Men (general population) |  |  | 74.3% |
| Deatsman et al., 2016 | Women seeking obstetric/gynaecologic care |  |  | 72.6% |
| Ekelin et al., 2012 | Students |  | Gonorrhoea: 37% women, 40% men | Chlamydia: 70% women, 65% men |
| Hammarberg et al., 2016 | Nurses |  |  | 83% |
| Heywood et al., 2016 | Students |  |  | 76.3% |
| Ikimalo & Babatunde, 2012 | General population |  |  | 88.7% |
| Iliyasu et al., 2013 | General population | 30.5% |  |  |
| Lundsberg et al., 2014 | General population |  |  | 69.4% |
| Mogilevkina et al.,2016 | Medical students | STI affect female fertility: 22% men  STI affect male fertility: 39% women; 21 men | STI affect female fertility: 42% women |  |
| Pitts & Hanley, 2004 | Students |  |  | 66.6% |
| Quach & Librach, 2008 | Students |  | Women: 59.9%, men: 55% |  |
| Switf & Liu, 2014 | Women attending fertility clinic |  |  | Chlamydia/gonorrhoea: 60.7% |
| Trent et al., 2006 | Adolescents |  |  | Women: 81.9%, men: 77.9% |
| Tyden et al., 2006 | Students | STI’ s and other disesases and infections: 30% |  |  |
| Smoking, alcohol, drugs, weight, stress as risk factors (n=30) | | | | |
| Abolfotouh, et al., 2013 | Patients seeking fertility care | Alcohol: outpatients: 15.7% | alcohol: IVF patients: 53.9%,  smoking: IVF patients: 51%, outpatients: 46.5% | psychological distress; 72.1% ; 58.7% |
| Ali et al., 2011 | General population | Smoking: 24% |  | Psychological stress: 65% Obesity: 58% |
| Bavan et al., 2011 | Students |  |  | Smoking: 74% |
| Bunting & Boivin, 2008 | Students |  |  | age, smoking, weight, drinking, stress, STD, drugs: 91% |
| Chelli et al., 2015 | Midwifery students |  |  | Smoking: 84.9% |
| Childress et al., 2015 | Women attending first fertility visit |  | M=59.4%; SD=16.5% |  |
| Conceição et al., 2017 | University students |  |  | Risk factors to male fertility: M=6.30; SD=1.62 (range 0-11)  Risk factors to female fertility: M=6.86; SD=1.94 (range 0-11) |
| Daniluk & Koert, 2013 | Men (general population) |  | Weight: 48.4% | Smoking cigarettes/marijuana: 77.4% |
| Daniluk& Koert, 2015 | General population |  |  | Weight: 72.9%  Smoking cigarettes/marijuana:88.9% |
| Daniluk et al., 2012 | Women (general population) |  |  | Weight: 66.2% |
| Daumler et al., 2016 | Men (general population) |  | Obesity: 54.2% | Stress: 68.9%  Smoking: 67.8%  Drugs: 64.6%  Alcohol(>10 drinks/week): 63.2 |
| Deatsman et al., 2016 | Women attending gynaecology/obstetric clinics |  |  | Smoking: 68.4%  Obesity: 69.5%  Alcohol use:67.4% |
| Ekelin et al., 2012 | Students |  | Obesity: 53% men  Stress: 53% men | Obesity: 87% women  Low weight: 73% women, 44% men  Smoking: 62% women, 75% men  Alcohol: 63% women, 65 men  Drugs: 81% women, 85% men  Stress: 63% women |
| Fotopoulou et al., 2015 | Medical students |  |  | Smoking: 91.2% |
| Fugener et al., 2013 | General population | M fertility risks mentioned affecting female: 2.84 of 14; M fertility risks mentioned affecting male: 2.60 of 14 |  |  |
| Hammarberg et al., 2016 | Nurses | Smoking for women: 24%  Passive smoking for women: 36% |  | Passive smoking for men: 89%  Obesity for women: 94%  Obesity for men: 75% |
| Hammarberg et al., 2013 | Women general population | Male obesity: 30%  Male smoking:36% | Female obesity and smoking: 59% |  |
| Heywood et al., 2016 | Students |  | Overweight: 54.5%  Underweight: 52.5% | Drugs: 75.3%  Alcohol: 72.1%  Smoking: 69.1% |
| Homan & Norman, 2009 | Patients seeking fertility care |  |  | Smoking: 90% women, 70% men  Being over or underweight: 100% women, 70% men  Alcohol: 60% women, 60% men  Psychological stress: 70% females, 70% males  Recreational drugs: 80% females; 90% males |
| Ikimalo & Babatunde, 2012 | General population |  |  | Excessive alcohol consumption: 90%  Excessive smoking: 90% |
| Lundsberg et al., 2016 | Women general population |  |  | Alcohol: 69.3%  Smoking: 71.3%  Being underweight: 73.2%  Being obese: 74%  Stress: 90% |
| Machado et al., 2014 | Students | Female Smoking: 19%  Male smoking: 15.9%  Obesity: 18%  Alcohol:16.8% |  |  |
| Maheshawari et al., 2008 | Sub fertile women and pregnant women |  |  | 93% subfertile, 88% pregnant |
| Meissner et al., 2016 | Students |  |  | Risk factors: 77% |
| Mogilevkina et al., 2016 | Medical students | Obesity affects female/male fertility: 1% women, 0.5% men |  | Smoking, alcohol, drugs affects female fertility: 67% women, 68% men  Smoking, alcohol, drugs affects male fertility: 67% women, 66% men |
| Nouri et al., 2014 | Students | Smoking: 35% medical students, 29% non-medical students | Alcohol: 54% non-medical students  Overweight: 56% non-medical students | Alcohol: 65% medical students  Overweight: 72% medical students  Underweight: 92% medical students, 88% non-medical students |
| Pitts & Hanley, 2004 | Students | Smoking: 23.6% |  |  |
| Quach & Librach, 2008 | Students | Being underweight or overweight: 39.1% men | Being underweight or overweight: 47.3% women | Smoking: 66% women, 66.7% men |
| Swift & Liu, 2014 | Women attending fertility clinic |  |  | Overweight: 78.6%  Stress: 95.6%  Smoking >10 cigarettes/day: 88.6% |
| Tyden et al., 2006 | students | Stress: 33%; Alcohol/drugs: 37% | Smoking: 46% |  |
| Note: When referred, women and men, it means the percentage of correct answers gave by women and men. | | | | |

| **Table B.5. Studies assessing awareness about the fertile period.** | | | | |
| --- | --- | --- | --- | --- |
| **Authors, year** | **Sample** | **Low (<40%)** | **Average (40-59%)** | **High (≥60%)** |
| Ali et al., 2011 | General population |  | 46% |  |
| Bennett et al., 2015 | Patients seeking fertility treatment |  |  | 70% |
| Bloom et al., 2000 | Men | 21.4% |  |  |
| Garcia et al., 2015 | Women candidate to oocyte donation |  | 50.7% |  |
| Hammarberg et al,, 2016 | Nurses |  |  | 75% |
| Hammarberg et al., 2013 | Women | 32% |  |  |
| Hashim et al., 1994 | Expectant mothers | 34% |  |  |
| Lundsberg et al., 2014 | Women |  |  | 60% |
| Ozsoy et al., 2012 | Students |  | 40% |  |
| Swift & Liu, 2014 | Women attending fertility clinic |  |  | 76.4% |
| Uddin & Choudhury, 2008 | Adolescent women | 9% |  |  |

| **Table B.6. Studies assessing awareness regarding the consequences of delaying childbearing.** | | | | |
| --- | --- | --- | --- | --- |
| **Authors, year** | **Sample** | **Low (<40%)** | **Average (40-59%)** | **High (≥60%)** |
| Behboudi-Gandevani et al., 2013 | Prim gravid women | Increased probability of to have a multiple birth after 35 years: 25%;  Increased probability to have a caesarean section after 35 years: 27%;  Increased probability to have a preterm baby after 35 years: 31%;  Increased probability to have a low birth weight after 35 years: 24%;  Increased probability of having a stillbirth after 35 years: 13% | Increased probability of having a baby with Down syndrome after 35 years:51%;  Increased probability of having a baby with a congenital anomaly after 35 years: 41%;  Increased probability to develop hypertension during pregnancy after 35 years: 53% | Increased probability of having problems becoming pregnant after 35 years:61%;  Increased probability to develop gestational diabetes after 35 years:61% |
| Bretherick et al., 2010 | Students | Age increased the risk of miscarriage: 25% |  |  |
| Daniluk & Koert, 2013 | Men (general population) | Children born to fathers >45 years have higher rates of learning disabilities, autism, schizophrenia and some cancers: 27.1% |  | Rates of miscarriage are higher for women in their 40s than for women in their 30s:69.2% |
| Daniluk & Koert, 2015 | General population | Children born to fathers >45 years have higher rates of learning disabilities, autism, schizophrenia and some cancers: 33% |  | Rates of miscarriage are higher for women in their 40s than for women in their 30s:82% |
| Daniluk et al., 2012 | Women (general population) |  |  | Rates of miscarriage are higher for women in their 40s than for women in their 30s: 83.8% |
| Deatsman et al., 2016 | Women attending obstetric /gynaecologic care |  |  | Awareness of health risk in pregnancy over 35 years:  Diabetes: 69%;  Miscarriage: 80%;  High blood pressure: 76%;  Genetic abnormalities: 75% |
| Gossett et al., 2013 | General population |  |  | women’s awareness of the implications  of advanced maternal age for pregnancy and fertility: AMA knowledge score, range = 0–7; mean=5.8; SD=1.3) |
| Guedes et al., 2014 | General population (pregnant couples) | Multiple pregnancy: women: 5.30%; men:6.30%  Low birth weight: women:16.805; men: 24.20%  Stillbirth: women: 27.40%; men: 30.50%  Gestational diabetes: women:24.20%; men:37.90%  Caesarean: women: 16.80%; men:17.90% | Preterm birth: women: 40%; men: 46.30% | Down syndrome: women: 83.20%; men: 83.20%  Medical help to conceive: women:61.10%; men: 60% |
| Lundsberg et al., 2014 | Women (general population) |  |  | Miscarriage: 79.3%  Genetic/chromosomal abnormality: 77.7% |
| Machado et al., 2014 | Students | Postponing is related with higher-risk pregnancy: 32% |  |  |
| Maheshwari et al., 2008 | Subfertile and pregnant women | Increased probability of having twins with age: 20% subfertile, 22% pregnant women | Increased probability of gestational diabetes with age: 48% subfertile, 41% pregnant women;  Increase probability of cesarean section with age: 46% subfertile, 41% pregnant women | Increase probability of miscarriage with age: 72% subfertile, 65% pregnant;  Increased probability of Down syndrome: 86% subfertile, 85% pregnant;  Increased probability of high blood pressure during pregnancy: 71% subfertile, 69% pregnant |
| Tough et al., 2006 | Woman with children | Increased probability of to have a multiple birth after 35 years:24%  Increased probability to have a caesarean section after 35 years:19%  Increased probability to have a preterm baby after 35 years: 22%  Increased probability to have a low birth weight after 35 years:11% | Increased probability of having a baby with a congenital anomaly after 35 years: 53%  Increased probability of having a low birth weight in women <20 years compared to women 20-29 years: 44% | Increased probability of having problems becoming pregnant after 35 years:85%  Increased probability of having a baby with Down syndrome after 35 years:86%  Increased probability of having medical problems during pregnancy after 35 years: 72%  Women >35 years are eligible to amniocentesis during pregnancy: 83% |
| Tough et al., 2007 | Childless women and men | Increased probability of to have a multiple birth with age: women: 15%; men: 11%  Increased probability to have a preterm baby with age: women: 39.7%; men: 37%  Risk of stillbirth varies with a age: women: 32%; men: 36% | Increased probability of having a baby with Down syndrome with age: men: 52%  Increased probability of having a low birth weight in women <20 years compared to women 20-29 years: women: 52%; men: 44%  Gestational diabetes: 48% men  Increased probability to have a caesarean section with age: women: 41%; men: 44% | Increased probability of having a baby with Down syndrome with age: women: 74%  Increased probability of having problems becoming pregnant with age: women: 78%; men: 72%  Gestational diabetes: women: 63%  Increased probability of having problems becoming pregnant with age: women: 74%; men: 65% |
