# Supplementary material for: What do people know about fertility? A systematic review on fertility awareness and its associated factors
Source: Ups J Med Sci. 2018 Jun 29;123(2):71–81. doi: 10.1080/03009734.2018.1480186 (PMC6055749; doi:10.1080/03009734.2018.1480186)
Supplement: Supplemental Material [file IUPS_A_1480186_SM6860.zip › Pedro_supp_matt/Supplemental_Table_A.1.docx]

| What Do People Know about Fertility? A Systematic Review on Fertility Awareness and its Associated Factors  Juliana Pedro*, Tânia Brandão, Lone Schmidt, Maria E. Costa and Mariana V. Martins  Supplemental Data A.1.  Table A.1. Main characteristics and findings of all studies included in this review. | | | | | | |
| --- | --- | --- | --- | --- | --- | --- |
| Authors, Year | **Country** | **Type of study, population and sample size** | **Measure used** | **Mean age,**  **Mean years of education** | **Reproductive and relationship status** | **Main findings:**  **Fertility awareness level (based on percentage of correct answers)** |
| Abiodun et al., 2016 | Nigeria | CROSS SECTIONAL  239 undergraduate students 231 f, 158 m | The Awareness of fertility issues (Lampic et al., 2006) | M age: 18.74 (SD=2.14) ; mean number of years at university: 2.54 (SD= 1.07) | 81.7% were single; 14.7% were in a committed relationship; 2.3% engaged and 1.3% married. More than 99% did not have children | Age-related fertility decline: low  Most fertile age: moderate |
| Abolfotouh, et al., 2013 | Saudi Arabia | CROSS-SECTIONAL  104 patients seeking fertility care (67f, 37 m) and 269 outpatients visiting the same hospital (31f, 238m) | Interview questionnaire based on Ali et al., 2011 | IVF patients: M age: 33.84 (SD=6.06); education level: 61.5% have secondary level or more; Outpatients: M age: 35.84 (SD=11.13);  education level: 69.9% had secondary level or more |  | Causes of infertility: moderate to high  Misconceptions: high **  Risk factors: moderate  Age as risk factor: moderate |
| Adashi et al., 2000 | Belgium, France, Germany, Italy, Sweden, UK, USA, Australia | CROSS-SECTIONAL  7036 adults from Europe and USA and 1158 from Australia | Study-specific telephone interview | adults aged > = 15 years in USA and Europe and >18 in Australia |  | Infertility definition: moderate  Age-related fertility decline: low |
| Ali et al., 2011 | Pakistan | CROSS SECTIONAL  457 individuals that were accompanying patients to hospital (246 f, 201 m) | Study-specific interview questionnaire | M age: 36 years old (SD=13 years);  46% had > 10 years of education; | 74% were married | Infertility definition: low  Causes of infertility (both male/female and causes): moderate to high  Misconceptions: moderate to high **  Risk factors: moderate  Fertile period: moderate |
| Al Khazrajy & Al Abayechi, 2009 | Iraq | CROSS-SECTIONAL  203 Infertile male patients attending fertility center | Study-specific questionnaire | M age: 35.67 years old |  | Infertility definition: moderate  STI as risk factor: high  Age as risk factor: moderate |
| Bavan et al., 2011 | USA | CROSS-SECTIONAL  328 female university students | Reproductive Health Education Quiz (Bavan et al., 2011) | M age: 22 years old (SD=2.5);  Education: 67% some college; 25% 4 year college, 7% master degree, 1 doctorate degree | 51% were currently in a relationship M = 2.3 years (SD = 1.9) | Misconceptions: moderate**  Risk factors: high  Age-related fertility decline: moderate |
| Behboudi-Gandevani et al., 2013 | Iran | CROSS-SECTIONAL  675 Prim gravid pregnant women | Maternal Age-related Risks of Childbearing (Tough et al., 2006) | M age: 36.87 years old;  12.6 years of education |  | Consequences of delaying childbearing: low to moderate |
| Bennett et al., 2015 | Indonesia | CROSS-SECTIONAL  212 female seeking fertility treatments | Study-specific interview | M age: 31 years old; 60% had a bachelor or master degree |  | Infertility definition: moderate  Causes of infertility (both male/female): high  Fertile period: high |
| Bloom et al., 2000 | India | CROSS-SECTIONAL  6727 men | Study-specific questionnaire | Age range: 15-59 years old; most frequent age group: 25-34 years old (32%) | Married men | Fertile period: low |
| Bretherick et al., 2010 | Canada | CROSS-SECTIONAL  360 female undergraduate students | study-specific questionnaire | M age: 21.28 years old (SD=2.81) | 88.8% single | Age as risk factor: moderate  Age-related fertility decline: low to moderate  Consequences of delaying childbearing: low |
| Bunting & Boivin, 2008 | United Kingdom | CROSS-SECTIONAL  149 (110 f and 39 m) postgraduate and undergraduates students | FAFS questionnaire (Bunting & Boivin, 2008) | M age: 24.01 years old (SD=7.81) |  | Misconceptions: high**  Risk factors: high |
| Bunting et al., 2013 | 79 countries | CROSS-SECTIONAL  10045 participants (8355 f, 1690 m) | Cardiff Fertility Knowledge Scale, CFKS (Bunting et al., 2013) | M age: 31.8 years old; 53.9% had university education | Married/living with partner for 5.9 years (SD=4.2); trying to conceive 2.8 years (SD=2.9) | General fertility awareness: moderate |
| Chan et al., 2015 | China | CROSS-SECTIONAL  367 university students (275 f; 92 m) | The Awareness of fertility issues (Lampic et al., 2006) | M age: 23.2 years (SD = 3.5);  53% in the first year of university | 58% single; 35% have a romantic partner; 7% married; 99% did not have children | Most fertile age: low  Age-related fertility decline: low |
| Chelli et al., 2015 | France | CROSS-SECTIONAL  285 midwifery students | Reproductive Health Education Quiz (Bavan et al., 2011) | M age: 22.6 years (SD=1.9) | Some have children; 62.8% have a partner; 61.8% would consider a pregnancy project | Risk factors: high  Age-related fertility decline: low |
| Childress et al., 2015 | USA and others | PROSPECTIVE SURVEY (PRE-POSTTEST STUDY)  234 Women seeking fertility treatment | FERT-AP (Childress et al., 2015) | M age: 34.8 years old (SD=4.7); education: more than 90% had a >=4 year college degree | Patients; 79.9% married; 82.9% without children | General fertility awareness: moderate to high |
| Conceição et al., 2017 | Portugal | PRE-POST INTERVENTION STUDY  173 university students (140f, 33 m) | The Awareness of fertility issues (Lampic et al., 2006) | M age: 20.18 (SD=4.93); 94.8% in the first and second year of university | Had children: 2.89%; 49.13% had a romantic relationship; 94.22% are single | Infertility definition: moderate  Risk factors: high  Most fertile age: low  Age-related fertility decline: low to moderate |
| Daniluk & Koert, 2013 | Canada | CROSS-SECTIONAL  599 men | Fertility Awareness Survey (Daniluk et al., 2012) | M age:33.9 years old (SD=8.99); 64.7% had college or university degrees | Childless men; 53.2 % married | Causes of infertility (both male/female and causes): moderate  Misconceptions: moderate**  STI as risk factor: high  Risk factors: moderate to high  Age-related fertility decline: moderate  Consequences of delaying childbearing: low to moderate |
| Daniluk & Koert, 2015 | Canada | PRE-POST INTERVENTION STUDY  199 participants (151f, 48m) | Fertility Awareness Survey (Daniluk et al., 2012) | M age: 28 years old; 67.3% completed college/university degrees | 52.8% single; 45.2% married | Causes of infertility (both male/female and causes): low  Misconceptions: moderate**  STI as risk factor: high  Risk factors: high  Age as risk factor: moderate  Age-related fertility decline: moderate  Consequences of delaying childbearing: low to moderate |
| Daniluk et al., 2012 | Canada | CROSS-SECTIONAL  3345 Women | Fertility Awareness Survey (Daniluk et al., 2012) | M age: 29 years old (SD=6.6);  65.6% have completed college/university degrees | Childless women; 53% married or living together; 34.2% single | Causes of infertility (both male/female and causes): moderate  STI as risk factor: high  Misconceptions: moderate**  Risk factors: high  Age as risk factor: moderate  Age-related fertility decline: moderate  Consequences of delaying childbearing: high |
| Daumler et al., 2016 | Canada | CROSS-SECTIONAL  701 men | Study-specific questionnaire | M age = 34.1 (SD = 9.1) | 49% married or living together; 60.1% childless | Causes of infertility: moderate  STI as risk factor: high  Risk factors: high  Age as risk factor: high |
| Deatsman et al., 2016 | USA | CROSS-SECTIONAL  94 women attending obstetrics/gynaecology clinic | Study-specific questionnaire | M age: 30.9 years old (SD=9.2) | 65.3% had children; 18.1% present difficulties in becoming pregnant; 8.5% required fertility treatment | STI as risk factor: high  Risk factors: high  Age as risk factor: high  Age-related fertility decline: low  Consequences of delaying childbearing: high |
| Ekelin et al., 2012 | Sweden | CROSS-SECTIONAL  247 students (101 f, 146 m) | The Awareness of fertility issues (Lampic et al., 2006) | 92.3% were between 18 and 20 years old | Childless students | STI as risk factor: moderate  Risk factors: high  Most fertile age: high  Age-related fertility decline: low |
| Fotopoulou et al., 2015 | Greece | CROSS-SECTIONAL  422 Female medical students | Reproductive Health Education Quiz (Bavan et al., 2011) | M age: 20 years (SD=1.75) | 2 students had children; 60% desired to get pregnant in next 5 to 10 years | Risk factors: high  Age-related fertility decline: low |
| Fugener et al., 2013 | Germany | CROSS-SECTIONAL  498 young people (306 f, 192 m) | FAFS questionnaire (Bunting & Boivin, 2008) | Mean age=22.04 years Old; SD=2.5  30.7% medical students, 38.2% students from other faculties, 31.1% vocational trainees |  | Risk factors: low  General reproduction awareness: low |
| Fulford et al., 2013 | 38 countries | CROSS-SECTIONAL  1345 Women | Cardiff Fertility Knowledge Scale, CFKS (Bunting et al., 2013) | M age=28.5 years old (SD=5.6); majority have university education | Childless women living with partner (M=3.8; SD=3); trying to conceive (M=1.5 years; SD=1.9) and never engaged in fertility treatments | General fertility awareness: moderate |
| Garcia et al., 2016 | Spain | RANDOMISED CONTROLLED TRIAL  201 women candidate to oocyte donation | Study-specific structured interview(Garcia et al., 2015) | M age=25.3 years old (SD=4.7); 31.3% had university education | 64.7% were childless | General fertility awareness: low |
| Garcia et al., 2017 | Spain | CROSS-SECTIONAL STUDY  201 professionals (gynaecologists, physicians and nurses) | Study-specific structured interview (Garcia et al., 2015) | M age = 42.7 (SD = 11.0) | 60.5% have children | Age-related fertility decline: moderate |
| Garcia et al., 2015 | Spain | CROSS-SECTIONAL STUDY  229 women candidate to oocyte donation | Study-specific questions based on Bunting et al., 2008, Lampic et al., 2006, Virtala et al., 2011 | M age=24.6 (SD=4.8); 48% had secondary education | 57.2% reported to have a stable partner; 63.3% had no children | Most fertile age: moderate  Age-related fertility decline: low  Fertile period: moderate |
| Gossett et al., 2013 | USA | CROSS-SECTIONAL  300 women seeking gynaecologic care | TKS (Gossett et al., 2013) | M age=34.8 (SD=7.9);46% had completed 4-year college degree | Healthy women (non-pregnant and not seeking fertility treatment); 54.6% married; 70% had been pregnant before | Consequences of delaying childbearing: high |
| Guedes & Canavarro, 2014 | Portugal | LONGITUDINAL STUDY  95 Couples at prenatal diagnosis visit aged 35 years | Maternal Age-related Risks of Childbearing (Tough et al., 2006) | M age women=37.08 (SD=2.43); M age men=37.24 (SD=5.30);  72.6% of women and 50.5% of men had a college degree | 10.5% had previous children; 26.3% had undergone prior fertility treatment | Age-related fertility decline: moderate  Consequences of delaying childbearing: low to moderate |
| Hammarberg, 2016 | Australia | CROSS-SECTIONAL STUDY  102 (100 f, 2 m) nurses working in general practice and other primary health care settings | Study-specific telephone interview | 19% is <35 years old; 82% is >35 and <66 |  | Causes of infertility: moderate  STI as risk factor: moderate  Risk factors: high  Age-related fertility decline: low  Fertile period: high |
| Hammarberg et al., 2013 | Australia | CROSS-SECTIONAL STUDY  462 Women and men | Study-specific questionnaire | M age:27.30 (SD=6.79); 13% had 13% or less years of schooling; 37% completed secondary school; 28% had a post-school diploma; 18% had a university degree | 49% were married;39% not in a relationship; 11% in a relationship but not living together; 30% have 1 or more children | Risk factors: low to moderate  Age-related fertility decline: low  Fertile period: low |
| Hashiloni-Dolev et al., 2011 | Israel | CROSS-SECTIONAL STUDY  410 undergraduate students (300 f, 108 m) | Awareness of age-related fertility decline questionnaire (Hashiloni-Dolev et al., 2011) | M age: 24.1 years old (SD 3.7) | 91.9% were single; 3.4% had already children | Age-related fertility decline: low |
| Hashim, 1994 | Saudi Arabia | CROSS-SECTIONAL STUDY  100 expectant mothers | Study-specific questionnaire interview | M age = 26.6 years; 26% had completed college or university | 78.3% had 2 or more children | Fertile period: low |
| Heywood et al., 2016 | Australia | CROSS-SECTIONAL STUDY  1780 secondary school Students (1125 f, 655 m) | Study-specific questionnaire | Students were in 10 (42.5%), 11 (30%) and 12 (27.6%) year | Without children | STI as risk factor: high  Risk factors: moderate to high |
| Hodes-Wertz et al., 2013 | USA | CROSS-SECTIONAL STUDY  183 women who completed >1 cycle of cryopreservation for non-medical reasons | Study-specific questionnaire | M age: 39 years old; (SD=2.7) | 77% were never married; 84%were in a romantic relationship | Most fertile age: high  Age-related fertility decline: moderate |
| Holton et al., 2016 | Australia | CROSS-SECTIONAL STUDY  1104 men of reproductive age | Study-specific questionnaire | M age = 36.3 | 53.4 were married; 61.1% had post-secondary education | Age-related fertility decline: moderate |
| Homan & Norman, 2009 | Australia | CROSS-SECTIONAL STUDY  20 patients (10 couples) seeking fertility care | Study-specific interview | M age (women): 36.2 years old; M age (men):37.1 years old. | 80% were childless | Risk factors: high |
| Ikimalo & Babatunde, 2012 | Nigeria | CROSS-SECTIONAL STUDY  150 adults (60 f, 90 m) | Study-specific questionnaire | Age group: 20-29: 70%; 30-39: 16%;40-49: 12.7%;50-59: 1.3%  Education: None: 5.3%; Secondary: 28%;Tertiary: 66.7% | Marital status:  Single: 58.7%; Married: 29.3%; Separated: 5.3%; Live-in: 6.7% | Infertility definition: low  Causes of infertility (both male/female and causes): moderate  Misconceptions: low**  STI as risk factor: high  Risk factors: high |
| Iliyasu et al., 2013 | Nigeria | CROSS-SECTIONAL STUDY  581 adults  (291 f, 290 m) | Study-specific questionnaire based on Adashi et al. (2000) | M age = 35.3 years old (SD = 10.5)  Education: Non-formal: 22%; Primary: 19.1%; Secondary: 30.1% Tertiary: 28.7% | Number of children:  0: 18.8%  1-5: 43.4%  > 6: 37.8% | Infertility definition: low  Causes of infertility (both male/female and causes): low to moderate  Misconceptions: moderate**  STI as risk factor: low |
| Lampic et al., 2006 | Sweden | CROSS-SECTIONAL  401 university students (222 f, 179 m) | The Awareness of fertility issues (Lampic et al., 2006) | Female M age: 23.9 years old (SD = 4.1)  Male M age: 23.7 years old (SD = 3.7) | Stable relationship: Female 60% yes, Male 51% yes  Have children: Female 9%, Male 5%: | Most fertile age: moderate to high  Age-related fertility decline: low |
| Lucas et al., 2015 | New Zealand | CROSS-SECTIONAL STUDY  683 university students  453 f, 226 m | Awareness of age-related fertility decline questionnaire (Hashiloni-Dolev et al., 2011) | M age: 22.3 years old (SD = 4.94) | Marital status: Married - 7%, Partner - 23%, Single - 69%, Did not specify - 0.06%  Already a parent: Yes - 5.27%, No - 93.7%, Did not specify - 1.02% | Age-related fertility decline: low |
| Lundsberg et al., 2014 | USA | CROSS-SECTIONAL  1000 women of reproductive age | Study-specific questionnaire | Age groups: 18-24: 163 women, 25-34: 592 women, 35-40: 245 women  Education: 20.1% high school or less, 79.4% some college or more | Relationship status:15% single and not dating, 6.3% single and dating, 10% in a relationship, 17% cohabiting, 51.4 married | Causes of infertility: moderate  STI as risk factor: high  Risk factors: high  Age as risk factor: high  Fertile period: high  Consequences of delaying childbearing: high |
| Machado et al., 2014 | Portugal | CROSS-SECTIONAL STUDY  3585 university students (76.9% were female) | Study-specific questionnaire | M age = 23.7 years old (SD = 5.7) |  | Risk factors: low  Age as risk factor: low  Consequences of delaying childbearing: low |
| Maeda et al., 2015 | Japan | CROSS-SECTIONAL STUDY  4328 participants (2164f; 2164 m)  618 triers: (309f, 309m (i.e., currently  trying to conceive for at least 6 months) | Cardiff Fertility Knowledge Scale, CFKS (Bunting et al., 2013) | General group: aged 18-59; M age = 39.3 (SD = 11.2)  Triers group: aged 18-50; M age = 35.2 (SD = 6.9)  University education: General group: 42.8%; Triers group: 46.9% | Given birth / fathered child: yes - general group 49.5%; triers 39.2%  Currently trying to conceive: general group 12.8%  Prior medical consultation for fertility: triers group 34.8%  Number of years trying to conceive: M = 2.9 (SD = 5.4) | General fertility awareness: moderate |
| Maeda et al., 2016 | Japan | RANDOMISED CONTROLLED TRIAL  1455 participants (729f, 726 m) hoping to have children | Cardiff Fertility Knowledge Scale, CFKS (Bunting et al., 2013) | Male Intervention group M age = 30.9 (SD = 5.8); Control group1 M age = 30.8 (SD = 5.7); Control group2 M age = 30.9 (SD = 5.7)  Female Intervention group M age = 30.5 (SD = 5.6); Control group1 M age = 30.4 (SD = 5.6); Control group2 M age = 30.2 (SD = 5.5) | Male Intervention group 55% university education; Control group1 57.9% university education; Control group2 52.5% education university; 50.8% single; 48.4% single; 43.4% single respectively.  Female Intervention group 41.2% university education; Control group1 32.5% university education; Control group2 45.7% education university; 29.2% single; 30% single; 30% single, respectively. | General fertility awareness: moderate |
| Maheshwari et al., 2008 | United Kingdom | CROSS-SECTIONAL STUDY  362 sub fertile women and 362 pregnant women | Study-specific questionnaire | Subfertile: M age = 32.60 (SD = 4.94); Pregnant: M age = 29.30 (SD = 5.57)  Subfertile: 35.1% had a university degree; Pregnant: 35.7% had a university degree | Subfertile group: Previous pregnancy: 42.5%  Pregnant group: Previous pregnancy: 60.2% | Risk factors: high  Age as risk factor: high  Age-related fertility decline: moderate to high  Consequences of delaying childbearing: moderate to high |
| Meissner et al., 2016 | Germany | CROSS-SECTIONAL STUDY  1144 university students; 77 % were female and 23 % were male | Study specific questionnaire based on Lampic et al., 2006; Bavan et al., 2011) | M age = 24.5 years old |  | Risk factors: high  Most fertile age: high  Age-related fertility decline: low |
| Mogilevkina et al., 2016 | Ukraine | CROSS-SECTIONAL STUDY  medical students:  (858 f, 407 m) | The Awareness of fertility issues (Lampic et al., 2006) | M age = 20.6 years old (SD = 2.4) | Have a steady partner: Females = 48%, Males = 41%  Had children: Females = 4%, Males = 2%  Experienced problems in becoming pregnant: Yes: Females = 2%, Males = 0.5%; No: Females = 7%, Males = 5% | Causes of infertility: low  STI as risk factor: low to moderate  Risk factors: moderate  Most fertile age: moderate  Age-related fertility decline: low |
| Mortensen et al., 2012 | Denmark | CROSS-SECTIONAL STUDY  863 female healthcare professionals | The Awareness of fertility issues (Lampic et al., 2006) | Median age = 32.6 years old (SD = 4.34), range 20 – 41  Education level:  Bachelor ’ s level (3 – 4 years) - 649 (75%) | Reproductive status: Currently pregnant 9%, Currently trying to get pregnant 12%  Have children: 48%  Relationship status: Single: 19%  In a relationship (incl. married): 81% | Most fertile age: high  Age-related fertility decline: low |
| Nouri et al., 2014 | Austria | CASE-CONTROL STUDY (CROSS SECTIONAL)  340 university students  (170 f, 170 m) | Study-specific questionnaire based on Rovei et al., 2010; Lampic et al., 2006) | M age: 20.03 years old (SD=1.77) years | Relationship status: single 75.3%, were in a relationship 24.7%, married or divorced: 0%  Had children: 1%. | Risk factors: moderate to high  Most fertile age: high  Age-related fertility decline: low |
| Ola et al., 2010 | Nigeria | CROSS-SECTIONAL STUDY  893 respondents (561f , 332 m) | Study-specific questionnaire | M age: 43.3 years | 60.5% married, 19.5% SINGLE; 65.7% literate | Causes of infertility (both male/female and causes): low  Misconceptions: low** |
| Ozsoy et al., 2012 | Turkey | CROSS-SECTIONAL STUDY  1291 students (674 f, 617m) | Study-specific questionnaire | M age = 22.7 years old(SD = 1.7) | Married – 25 (1.9%); Single – 1266 (98.1) | Fertile period: moderate |
| Petersen et al., 2015 | Denmark | CROSS-SECTIONAL  340 women from an infertility clinic | Study-specific questionnaire | M age = 37.4 years old (SD = 2.0)  Education: more than 4 years: 54.6% | 140 cohabiting; 200 single  85.3% were not trying to get pregnant | Most fertile age: high |
| Peterson et al., 2012 | USA | CROSS-SECTIONAL STUDY  246 undergraduate university students  138 f, 108 m | The Awareness of fertility issues (Lampic et al., 2006) | M age = 20.4 years (SD = 2.3)  Year in school: Women = 2.4 (SD = 1.1), Men = 2.5 (SD = 1.1) | 0% had a child  58% were single, 38% were in a committed relationship, 1% were married, 1% were engaged | Most fertile age: moderate  Age-related fertility decline: low |
| Pitts & Hanley, 2004 | Australia | CROSS-SECTIONAL STUDY  280 secondary students (160f, 120m) | Study-specific questionnaire | M age = 15.1 years old (SD = 0.74) |  | Causes of infertility: low to moderate  STI as risk factor: high  Risk factors: low  Age as risk factor: low |
| Quach & Librach, 2008 | Canada | DESCRIPTIVE STUDY (CROSS-SECTIONAL)  772 students (377f, 392m) | Study-specific questionnaire | M age: 17.5 years old (SD = 0.98)  Education: Grade 11: 28.57%; Grade 12: 71.43% |  | Infertility definition: high*( this study reported the percentage of people “familiar” with term infertility)  Causes of infertility (both male/female causes): high  STI as risk factor: moderate  Risk factors: moderate |
| Rovei et al., 2010 | Italy | EPIDEMIOLOGICAL STUDY (CROSS-SECTIONAL)  university students (607 f, 351 m) | Study-specific questionnaire | Age: M female= 22.1 years old (range 19–37); M male= 21.9 years old (range 19–34) |  | Most fertile age: high  Age-related fertility decline: low |
| Sorensen et al., 2016 | Denmark | CROSS-SECTIONAL  517 university students (438f, 79m) | The Awareness of fertility issues (Lampic et al., 2006) | Age: M female= 24.2 years old (SD=5.1); M male= 25.6 years old (SD=4.4) | Steady relationship: 60% men; 62% women  Had children: 8% men; 11% women  Pregnant at the time of the study: 1% men; 3% women | Most fertile age: moderate to high  Age-related fertility decline: low |
| Stern et al., 2013 | Sweden | RANDOMISED CONTROLLED TRIAL  201 women seeking contraceptive counselling, chlamydia testing and cervical screening | Study-specific questionnaire based on Lampic et al., 2006; Svanberg et al., 2006) | Intervention group (IG) and control group 1(CG1):  M age: IG: 23 years(SD=2.4); CG1: M age: 23 years (SD=2.2)  Education: IG: 73 had bachelor degree; CG1: 63 had bachelor degree | Relationship status:  IG: 58% in a stable relationship;42% single  CG1: 55% in a stable relationship: 45% single | General fertility awareness: low |
| Svanberg et al., 2006 | Sweden | CROSS-SECTIONAL STUDY  postgraduate students (141 f, 116 m) | Study-specific questionnaire | Age groups:  25-29 y: females: 55%, males: 56% | Stable relationship: Yes: females 87%; males 76%  (Have children: females: 25%, males 22%) | Age-related fertility decline: low |
| Stoebel-Richter et al., 2012 | Germany | CROSS-SECTIONAL STUDY  2110 participants (1181f, 929m) | Study-specific questionnaire | M age: 35.8 years (SD 9.1)  10th grade: female (49.9%), Male (41.5%) | 64.7% were in a committed relationship  60% had at least one child;  1.3% had had infertility treatment | Age-related fertility decline: low |
| Sugiura-Ogasawara et al., 2010 | Japan | CROSS-SECTIONAL STUDY  249 single women | Study-specific questionnaire | M age 25.2 years (SD=6.8) |  | Infertility definition: high*( this study reported the percentage of people “familiar” with term infertility) |
| Swift & Liu, 2014 | Canada | CROSS-SECTIONAL STUDY  140 women attending a fertility clinic | Study-specific questionnaire | The mean age of participants in the study was 34 years  (range 23 to 44)  Education: High school or less = 7.9%, College = 30.0%,  University undergraduate = 26.4%, University graduate = 23.6%,  University professional =12.1% |  | Causes of infertility: moderate  Misconceptions: moderate**  STI as risk factor: high  Risk factors: high  Age-related fertility decline: low  Fertile period: high |
| Tyden et al., 2006 | Sweden | CROSS-SECTIONAL STUDY  300 female university students | The Awareness of fertility issues (Lampic et al., 2006) | M age 23 years (range=19-37) | Stable relationship: 70%  Had been pregnant: 5%  Had children: 1% | Most fertile age: moderate  Age-related fertility decline: low  Risk factors: low |
| Tough et al., 2006 | Canada | CROSS-SECTIONAL STUDY  1,044 randomly selected women who delivered their first live-born infant (July 2002  and September 2003) | Maternal Age-related Risks of Childbearing (Tough et al., 2006) | Age:  <25=15.7%, 25-29=22.5%, 30-34=26.6%  35-39=31.7%, 40+ = 3.6%  Education: college or university undergraduate degree: 60.7% | Married: 93.3%  Used Assisted reproductive Technology: 12.2% | Consequences of delaying childbearing: moderate |
| Tough et al., 2007 | Canada | CROSS-SECTIONAL STUDY  500 men and 1006 women randomly selected without children (October 2003-February 2004) | Study-specific questionnaire based on Maternal Age-related Risks of Childbearing (Tough et al., 2006) | Mage female=31; Mage men=30  67% of women and 59% had post-secondary education | Married: 32.3% women, 47.8% men | Consequences of delaying childbearing: moderate |
| Trent et al., 2006 | USA | CROSS-SECTIONAL  302 adolescents  (166 f, 136m) | Study-specific telephone interview | M age: 14.7 years (14.7 for male (SD = 1.7), 14.6 for females (SD = 1.7)). | Fathered child/been pregnant: males:4%, females:11%  Sexual intercourse ever: males: 48%, females: 32%  History of STD: males: 5%, females: 5% | STI as risk factor: high |
| Uddin & Choudhury, 2008 | Bangladesh | CROSS-SECTIONAL STUDY  920 girls | Study-specific interview | M age: 14.4 (SD=2.9) | 45% had primary education; 52% of girls’ mothers do not have education | Fertile period: low |
| Vassard et al., 2016 | United Kingdom and Denmark | CROSS-SECTIONAL STUDY  women (n=1,000) and men (n=237) from the UK (40%) and Denmark (60%).  Data in fertility awareness: Females: 974-978; Males: 235 | Awareness of age-related fertility decline questionnaire (Hashiloni-Dolev et al., 2011) | Age: 18-24: 22%, 25-34: 43%, 35-44: 25%, +45:11%  Education: Undergraduate and postgraduate: 80% | Married: 45%, Cohabiting: 27%, Not cohabiting: 9%, Single: 19%  Have children: 48%, No children: 52% | Age-related fertility decline: moderate |
| Virtala et al., 2011 | Finland | CROSS-SECTIONALSTUDY  9967 undergraduate university students ( 3222f, 1864m) | The Awareness of fertility issues (Lampic et al., 2006) | M age male: 24.7 years;  M age female: 23.9 years | Stable sexual relationship: males 60.8%, females 69.8%.  Have children: males: 7.8%; females: 9.6%. | Age-related fertility decline: low |
| Note. M, mean; SD, Standard deviation; f, female; m, males; STI, sexually transmitted infections. ** Percentage of people believing in myths. | | | | | | |
