# Supplementary material for: What do people know about fertility? A systematic review on fertility awareness and its associated factors
Source: Ups J Med Sci. 2018 Jun 29;123(2):71–81. doi: 10.1080/03009734.2018.1480186 (PMC6055749; doi:10.1080/03009734.2018.1480186)
Supplement: Supplemental Material [file IUPS_A_1480186_SM6860.zip › Pedro_supp_matt/Supplemental_Table_A.2..docx]

**What Do People Know about Fertility? A Systematic Review on Fertility Awareness and its Associated Factors**

**Juliana Pedro*, Tânia Brandão, Lone Schmidt, Maria E. Costa and Mariana V. Martins**

**Supplemental Data A.2.**

| **Table A.2. Associations between FA and both sociodemographic and reproductive variables** | | | | | |
| --- | --- | --- | --- | --- | --- |
| Variable | Nr studies | **No association** | **Significant association** | | |
|  |  |  | **Positive** | **Mixed** | **Negative** |
| **Gender**  *(Positive association considered if women presented greater fertility awarenessthan men) | 26 | Abiodun et al., 2016^1^; Bunting et al., 2008; Chan et al., 2015^2^; Guedes & Canavarro, 2014; Hashiloni-Dolev et al., 2011^3^; Maeda et al., 2016; Nouri et al., 2014; Pitts & Hanley, 2004^4^; Quach & Librach, 2008^5^; Sorensen et al., 2016 | Ali et al., 2011*; Bunting et al., 2013*; Fugener et al., 2013*^6^; Hammarberg et al., 2013*; Heywood et al., 2016*; Maeda et al., 2015*; Meissner et al., 2016* ; Mogilevkina et al., 2016*; Stoebel-Richter et al., 2012*^7^; Vassard et al., 2016*; Virtala et al., 2011*; Tough et al., 2007* | Ekelin et al., 2012^8^; Lampic et al., 2006^9^; Peterson et al., 2012^10^; Rovei et al., 2010^11^ |  |
| **Age** | 23 | Bennett et al., 2015; Bunting et al., 2008; Daniluk & Koert, 2013; Daniluk et al., 2012; Daumler et al., 2016; Garcia et al., 2016; Hammarberg et al., 2016; Maeda et al., 2015; Mortensen et al., 2012^12^; Swift et al., 2014; Tough et al., 2007 | Ali et al., 2011; Bunting et al., 2013; Behboudi-Gandevani et al., 2013^13^; Garcia et al., 2015^14^; Gossett et al., 2013; Holton et al., 2016; Stern et al., 2013; Uddin & Choudhury, 2008; Virtala et al., 2011 | Bloom et al., 2000^15^; Lundsberg et al., 2014^16^ | Deatsman et al., 2016^17^ |
| **Education** | 24 | Daniluk & Koert, 2013; Garcia et al., 2015; Guedes & Canavarro, 2014; Heywood et al., 2016; Maeda et al., 2015^18^; Daumler et al., 2016 | Al Khazrajy & Al Abayechi, 2009; Behboudi-Gandevani et al., 2013; Bennett et al., 2015; Bloom et al., 2000; Bunting et al., 2008; Bunting et al., 2013; Childress et al., 2015; Daniluk et al., 2012; Garcia et al., 2016; Gossett et al., 2013; Hammarberg et al., 2013; Holton et al., 2016; Maeda et al., 2015^18^; Meissner et al., 2016; Stern et al., 2013; Stoebel-Richter et al., 2012^19^; Swift et al., 2014; Uddin & Choudhury, 2008; Tough et al., 2007 |  |  |
| Medical education /Health sciences (vs other education) | 6 | Rovei et al., 2010^20^ | Fugener et al., 2013^21^; Hashiloni-Dolev et al., 2011; Meissner et al., 2016; Nouri et al., 2014^22^; Garcia et al., 2017 ^23^ |  |  |
| **Reproductive status** | 20 |  |  |  |  |
| Infertile patients vs outpatients; participants requiring fertility treatment; patients who experienced prior infertility | 3 | Abolfotouh, et al., 2013; Guedes & Canavarro, 2014^24^ | Deatsman et al., 2016; Guedes & Canavarro, 2014^24^ |  |  |
| Trying to conceive; trying to conceive for more than 12 months; sub fertile vs pregnant | 2 |  | Maeda et al., 2015 | Maheshawari et al., 2008^25^ |  |
| Duration of infertility | 1 |  | Al Khazrajy & Al Abayechi, 2009 |  |  |
| Previous fertility treatmentt | 3 | Guedes & Canavarro, 2014^26^ | Gossett et al., 2013; Guedes & Canavarro, 2014^26^; Tough et al., 2006 |  |  |
| At least 1 child, number of children , not having children, have children; birth, adoption, stepchildren | 9 | Bunting et al., 2013; Garcia et al., 2015; Garcia et al., 2016; Guedes & Canavarro, 2014; Daumler et al., 2016 | Bloom et al., 2000; Childress et al., 2015 | Maeda et al., 2015^27^ | Holton et al., 2016^28^ |
| Desire to have children; currently planning a pregnancy | 3 | Hashiloni-Dolev et al., 2011^29^; Tough et al., 2007 | Hashiloni-Dolev et al., 2011^29^; Stern et al., 2013 |  |  |
| Plan to have children before the age of 30old | 1 | Lampic et al., 2006 |  |  |  |
| History of pregnancy; previous pregnancy | 4 | Stern et al., 2013; Mortensen et al., 2012^30^ | Deatsman et al., 2016 |  | Gossett et al., 2013 |
| Planned pregnancy | 2 |  | Behboudi-Gandevani et al., 2013; Tough et al., 2006 |  |  |

^1^ Significant gender differences were found for two of seven items.

^2^ Significant gender differences were found for one of seven items.

^3^ Significant gender differences were found for one of 12 items.

^4^ Gender differences were not significant for 17 of 20 items.

^5^ Gender differences were not significant for 11 of 12 items.

^6^ Significant gender differences were found for two of three subscales evaluated.

^7^ Significant gender differences were found for two out of three items.

^8^ Significant gender differences were found in three out of six items: women provided more accurate answers than men concerning age and marked fertility decline; men provided more accurate answers regarding the chance of pregnancy during ovulation and rate of infertile couples. Regarding risk factors, women answered significantly more often that overweight, underweight and age affect fertility, whereas men reported that smoking affects fertility more often.

^9^ Significant gender differences were found for six of eight items (women presented higher FA in four items and men presented higher FA in two items. Please note that gender differences were evaluated using the *X*^2^ statistic to compare male and female proportion of answers cross 4 categories of answers (multiple-choice questions).

^10^ Significant gender differences were found for four of eight items (women presented higher FA in two items; men presented higher FA in the other two items. Please note that gender differences were evaluated using the *X*^2^ statistic to compare male and female proportion of answers across four categories of answer (multiple-choice questions).

^11^ Significant gender differences were found for two of four items (these differences were not consistent).

^12^ Being less than 30 or more than 30 years old was not significantly associated with FA levels for seven of eight items.

^13^ Being more than 40 years old was significantly associated with greater FA.

^14^ Being 26-29 years old is significantly associated with greater levels of FA whereas being younger than 25 years old was not significantly related to FA levels.

^15^ Participants who were 35-44 years old presented significantly greater FA than 15-24, 25-34 and 45-59 age groups.

^16^Younger participants (18-24 years) presented less FA than the 25-34 and 35-40 age groups concerning infertility risk factors; 25- to 34-year-olds presented greater awareness than the 35-40 and 18-24 age groups regarding the effect of age on fertility; 35- to 40-year-olds presented greater knowledge than the 18-24 and 25-34 year age groups concerning ovulatory cycle and 35- to 40-year-olds presented significantly more misconceptions about fertility than the 25-34 and 18-24 year age groups.

^17^ Being less than 30 years old was significantly associated with greater awareness regarding age-related fertility decline. Regarding risk factors, no age differences were found (exception for one item).

^18^ University education was found positively associated with FA levels (in the general group); no significant association was found between university education and FA levels (in the “triers” group).

^19^ High education was significantly associated with greater FA for two of three items.

^20^ Three items in four did not show differences in FA between humanities and science students.

^21^ Medical students had greater FA levels.

^22^ In general, medical students had greater levels of FA. Female medical students had greater levels of FA than female non-medical students; however male medical students had similar FA kevels to male non-medical students.

^23^ Gynaecologists had greater FA levels than other physicians and nurses.

^24^ Male participants who had experienced prior infertility had greater FA, whereas this association was not found among females.

^25^ Significant differences in FA levels were found between subfertile and pregnant women in three of six items (the differences were not in the same direction).

^26^ Male participants who had experienced infertility treatments had greater FA, but this association was not found among women..

^27^ Giving birth was significantly associated with greater FA (in the general group), whereas in the “triers” group (i.e., people trying to conceive for a minimum 6 months) being a father was associated with less FA.

^28^ Being a father was significantly associated with less awareness regarding male fertility decline.

^29^ Participants who desired to have children had significantly greater FA regarding chances of becoming pregnant spontaneously, but they presented similar awareness regarding the chances of becoming pregnant via IVF.

^30^ Never had been pregnant vs ever pregnant was not significantly associated with FA levels in six of eight items.
